# Seroprevalence Study on West Nile Virus (WNV) Infection, a Hidden Viral Disease in Fars Province, Southern Iran

**DOI:** 10.18502/jad.v14i2.3735

**Published:** 2020-06-30

**Authors:** Masoumeh Amin, Morteza Zaim, Hamideh Edalat, Hamid Reza Basseri, Mohammad Reza Yaghoobi-Ershadi, Farhad Rezaei, Kourosh Azizi, Mostafa Salehi-Vaziri, Mohsen Ghane, Saideh Yousefi, Sorna Dabaghmanesh, Sedigheh Kheirandish, Mohammad Esmaeil Najafi, Jalal Mohammadi

**Affiliations:** 1 Department of Medical Entomology and Vector Control, School of Public Health, Tehran University of Medical Sciences, Tehran, Iran; 2 Department of Medical Entomology and Vector Control, School of Health, Shiraz University of Medical Sciences, Shiraz, Iran; 3 Department of Medical Virology, School of Public Health, Tehran University of Medical Sciences, Tehran, Iran; 4 Department of Arboviruses and Viral Hemorrhagic Fevers (National Ref Lab), Pasteur Institute of Iran, Tehran, Iran; 5 Department of Clinical Sciences, School of Veterinary Science, Shiraz University, Shiraz, Iran; 6 Department of Oral and Maxillofacial Pathology, Bushehr University of Medical Sciences, Bushehr, Iran; 7 Environmental Health Unit, Faculty of Health, Shiraz University of Medical Sciences, Shiraz, Iran

**Keywords:** West Nile virus, Seroprevalence, Iran

## Abstract

**Background:**

West Nile Virus, a mosquito-borne flavivirus, causes a variety of symptoms in human, from asymptomatic infection to neuroinvasive disease. Several studies have been conducted on the seroprevalence of WNV infection in different areas from Iran. This study was performed to find the presence of antiviral antibodies in human serum among some high risk population and awareness of health care staff about symptom of the WNV infection.

**Methods:**

Study performed in five geographical districts based on high population of immigrant and domestic birds and prevalence of the antiviral antibodies in horses which was reported previously. Totally 150 human blood samples were collected during 2018. The samples collected from patients referred to the clinics. The ELISA method used to detect IgG and IgM antibody against WNV. Logistic regression models used to analyze the effect of sex, age, keeping birds and urban/rural residence on the risk of infection. The awareness of health care staff about symptom of infection surveyed.

**Results:**

From all blood donors, 41 samples (27.33%) showed positive to IgG antibody. From which 56.10% were males and remaining females. None of the mentioned factors had a significant relationship. Health care staff had less attention to the infection.

**Conclusion:**

Although the prevalence of antibodies was relatively high, due to the similarity to other viral diseases, health care staff had less attention to the disease. The study showed that people in these areas have been exposed to the virus. Further research activities are recommended for control of this arbovirus.

## Introduction

West Nile Virus is a mosquito-borne virus that infects various mammal species especially horses and humans; however, the most commonly West Nile Virus infected animals are birds that serve as the reservoir host (1, 2). The birds show different signs of WNV infection as some of them become ill with high symptom of the disease and also die, while some others show no symptom. It is stated that house sparrows and crows are so susceptible to WNV infection but they are poor reservoir for transmission of virus via seems to be more responsible for conservation and transmission of the virus in United States (5). Furthermore, human and equines are incidental as well as dead-end hosts (6, 7).

The virus was first found from the blood of a woman in Northern Uganda in a region close to the Nile River in 1937. WNV is widespread in Africa, Europe, Asia, America and Caribbean Islands. In nature, birds are the reservoir of virus and mosquitoes get the virus from birds (8–10). The mosquitoes of the *Culex* species play an important role in the WNV transmission cycle (11–13). Humans become infected via mosquito bites. It can also get the virus through organ transplantation, breast feeding and blood transfusion. Human infections are mild or asymptomatic. Severe disease is stated in older patients. Most human infections have no signs (80%); nearly 20% of those infected develop a mild infection known as West Nile fever. Symptoms of the disease include vomiting, diarrhea, headache, backache, muscle aches, skin rash and enlarged lymph nodes. In severe cases, nervous system problems like inflammation of brain and spinal cord with hemorrhage, perivascular cuffing and neuronal degeneration have been detected. WNV has been measured a mild pathogen causing self-limiting outbreaks, but some newer isolates of virus seem to be lethal; therefore an increased incidence of neurological disease and a higher case fatality rate have been associated (4, 15, 16). Several studies have been carried out on the presence of antiviral antibodies in human serum in the world (17, 18, 19). These antiviral antibodies are Immunoglobulin M (IgM) and Immunoglobulin G (IgG). IgM is the first antibody to be immediately developed when any foreign particle is introduced. WNV-specific IgM antibodies are usually detectable 3 to 8 days after onset of illness and persist for 30 to 90 days, but longer persistence has been documented. Therefore, positive IgM antibodies occasionally may reflect a past infection. If serum is collected within 8 days of illness onset, the absence of detectable virus-specific IgM does not rule out the diagnosis of WNV infection, and the test may need to be repeated on a later sample. IgG is a long term response for any disease and thus protect our body from viral and bacterial attacks. WNV IgG antibodies generally are detected shortly after IgM antibodies and persist for many years following a symptomatic or asymptomatic infection. Therefore, the presence of IgG antibodies alone is only evidence of previous infection and clinically compatible cases with the presence of IgG, but not IgM, should be evaluated for other etiologic agents. The effect of IgM is temporary and they disappear after 2–3 weeks of their production. IgG is produced at the later stage of infection, but their effect is for a long time and helps in complete eradication of infections or diseases. Given that we only found IgG antibodies in the blood samples of people living in this study area, this indicates their contact with the virus in the past and possibly their WNV infection. IgM provides immediate response while IgG responds later with the permanent eradication of antigen and its effect is lasting. The amount of IgM produced at the time of exposure to antigen is six times greater than IgG (20–24). A study from 2008 to 2009 showed widespread circulation of WNV in Iran, mainly in southwestern provinces where the virus probably circulates every year (25). Previous seroepidemiological studies in Iran revealed the presence of WNV antibody in different regions of Iran (26–29). Apart from the study in Chabahar, South of Iran, no further study has been conducted in south of Iran (30). Although a study on the serum of horses in Fars Province has reported a high incidence of antibody against the virus (25), no coherent study has been carried out on the human infection in Fars Province, Southern Iran. Therefore, the aim of this study was to determine the presence of antiviral antibodies and previous exposure in human serum as well as of awareness of health staff and clinicians about symptom of the WNV. The results of current study demonstrate the primary information about situation of WNV in the intended area.

## Materials and Methods

### Geographical area and Date of Research

**Fig. 1.**
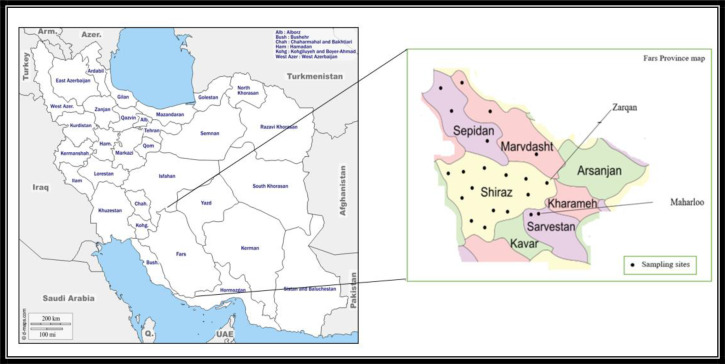
Location of study area and sampling sites in Fars Province, Southern Iran, 2018

This study was carried out in five selected geographic counties of Fars Province, Southern Iran during 2018 (Fig. 1). Fars Province is located in the South of Iran between 29°37′ N and 52°32′ E and covers an area of 122,400km^2^. The study areas include Shiraz, Zarqan, Marvdasht and Sepidan Counties as well Maharloo Wetland area. Generally, Shiraz district has urban texture and the habitants do not keep domestic animals at home. Therefore, the habitants are more common host for mosquito bites. In contrary, Marvdasht, Zarqan, Sepidan and Maharloo Counties have rural texture with many livestock and thus the mosquito vectors have more access to animal blood source rather than human. In addition, the criteria for chosen areas were presence of high population of immigrant and domestic birds as suspected reservoirs of WNV and high prevalence of antiviral antibodies in horses (according to previous study). Furthermore, the occurrence of suspected mosquito vectors in the area was considered (according to primary pilot in the current study).

### Awareness of Sign and Symptom about WNV

A cross sectional study was conducted which assessed awareness of sign and symptom of WNV disease among health care staff and practitioners of selected health facilities by using an open-ended questionnaire. The data were collected from 12 health centers.

According to data obtained from a student thesis at Shiraz Veterinary School (31), antibody titers were higher in horses in Shiraz, Zarqan, Marvdasht and Sepidan district than other areas of Fars Province. The Maharloo Wetland area, which hosts the largest number of migratory birds in the province, was also selected as one of the study areas. Three centers from Marvdasht, three centers from Sepidan, one center from Maharloo, two centers from Zarqan and three centers from Shiraz were selected. At these centers, all staff were asked about their knowledge of the symptoms of WNV infection.

### Serum Sample Collection

The serum samples were collected from patients who referred to the clinics of five selected areas during 2018. The sample size was 150 individuals. The desired parameters of each participant including age, gender, keeping birds and urban/rural residency were considered in a questionnaire. Five milliliter of venous blood was taken and the sera were stored at −20 °C until tested. All serum samples were referred to the Viral Laboratory, Department of Medical Virology, School of Public Health, Tehran University of Medical Sciences and were examined for antibody detection (IgG and IgM) against WNV.

### Enzyme Linked Immuno Sorbent Assay (ELISA)

This test was carried out by Euroimmune kit (EI_2662-9601G/M) manufactured in Germany to detect anti WNV antibodies (Immunoglobulin G and Immunoglobulin M) in the serum sample. The test was performed following the kit manufacturer’s protocol. The chosen kit was so specific without any cross activity to other Flaviviruses. The antigen source was a recombinant, detergent-extracted glycoprotein E of WNV from the membrane fraction of human cells (32).

**IgM capture Immunoassays:** Testing was performed according to the manufacturers’ instructions. Serum samples, along with the controls and cutoff calibrator, were diluted 1:100 in sample diluent and then added to microwells coated with anti-human IgM antibodies. Following a 1h incubation, the wells were washed and reconstituted recombinant WNV antigen was added. An incubation of 2h occurred, followed by a second wash and the addition of horseradish peroxidase (HRP)-conjugated mouse anti-flavivirus conjugate. After 30 min, a third wash step was employed, and a substrate consisting of tetramethylbenzidine (TMB) and hydrogen peroxide was added to each well. After 10min, 1Molar sulfuric acid was added to each well to stop the reaction, and the absorbance of each well was determined spectrophotometrically at 450nm (32).

**WNV IgG Enzyme Immunoassays:** Samples were diluted 1:100 in sample diluent and added to micro wells coated with recombinant WNV antigen and incubated for 60min. After the wells were washed, HRP-conjugated Fc fragment-specific anti-human IgG was added and the wells were incubated for 30min. A second wash was performed, and TMB substrate was incubated in the wells for 10min. To stop the reaction, 1Molar sulfuric acid was added to each well, and the absorbance of each well was determined spec-trophotometrically at 450nm (32).

### Statistical analysis

Logistic regression method (SPSS statistical package version 19.0; IBM SPSS Institute, USA) was used to evaluate significant differences in the seroprevalence positive rates by different demographic factors and a significance level of 0.05 was considered statistically.

### Ethical statement

The study suggestion was accepted by the Research Ethics Committee of the Tehran University of Medical Sciences, Tehran, Iran (No.IR.TUMS.SPH.REC.1396.4204) and informed written consent was obtained from the subjects for blood sampling.

## Results

The demographic characteristics of par ticipants are presented in Table 1. Overall, 150 volunteers participated in the study included 63 (42.00%) from urban and 87 (58.00%) from rural area. The majority of participants was from Shiraz County (40.00%) and the minimum of them was from Sepidan (13.33 %) followed by Zarqan (14.67%), Maharloo (16.00%) and Marvdasht district (16.00%). About one third of the participants (32.67%) stated that they have birds at home. The majority of participants belonged to the middle age group (30–50 years). Overall the prevalence of positive IgG antibody was detected among 27.33% participants (41 out of 150), which included 23 males (56.10%) and 18 females (43.90%). Among them, 27 (18.00 %) cases were from Shiraz County, 3 (2.00 %) were from Zarqan County, 4 (2.67%) cases were from Maharloo wetland area, 2 (1.33%) cases were from Sepidan County and 5 (3.33 %) cases were from Marvdasht County (Table 2). The prevalence of antibodies against WNV was not statistically significant by gender (P= 0.493).

**Table 1.** Demographic characteristics of participants from five districts, Fars Province, Southern Iran, 2018

**District**	**No.**	**Percent from total (%)**	**Gender**			

			**Male**	**Percent from total (%)**	**Female**	**Percent from total (%)**
Shiraz	60	40.00	31	20.66	29	19.33
Zarqan	22	14.67	12	8.00	10	6.67
Maharloo	24	16.00	12	8.00	12	8.00
Sepidan	20	13.33	10	6.67	10	6.67
Marvdasht	24	16.00	13	8.67	11	7.33
**Total**	150	100.00	78	52.00	72	48.00
**Age (year)**						
20–30	22	14.66	17	11.33	5	3.33
31–40	45	30.00	23	15.33	22	14.67
41–50	43	28.67	21	14.00	22	14.67
51–60	28	18.67	14	9.33	14	9.33
≥60	12	8.00	3	2.00	9	6.00
**Total**	150	100.00	78	52.00	72	48.00
Keeping birds	49	32.67	28	18.67	21	14.00
**Residence Place**						
Urban	63	42.00	34	22.67	29	19.33
Rural	87	58.00	53	35.33	34	22.67

**Table 2.** Frequency of positive West Nile virus IgG antibody according to the sex, Fars Province, Southern Iran, 2018

**Region**	**Number of Samples**	**Percent from total (%)**	**Positive Cases**			**Gender of positive cases**			**P-value**

			**No.**	**Percent from total (%)**	**Male**	**Percent from total (%)**	**Female**	**Percent from total (%)**	
**Shiraz**	60	40.00	27	18.00	15	36.58	12	29.26	
**Zarqan**	22	14.67	3	2.00	2	4.88	1	2.44	
**Maharloo**	24	16.00	4	2.67	1	2.44	3	7.32	
**Sepidan**	20	13.33	2	1.33	1	2.44	1	2.44	
**Marvdasht**	24	16.00	5	3.33	4	9.76	1	2.44	
**Total**	150	100.00	41	27.33	23	56.10	18	43.90	0.493

The positive cases based on the age group in study area are shown in Table 3. The infection rate to WNV among different age groups was so varied in different counties. None of the positive cases was found among 20–30 years old group in all counties, while the majority of positive case from Shiraz County occurred at age group of 31–40 followed by 41–50 year olds. Considering the age groups, the prevalence of antibody against WNV was not significantly different (P= 0.832). The positive cases based on keeping birds (as potent reservoirs) at home in five counties are shown in Table 4. The positive WNV IgG antibody was categorized based on keep birds as suspected reservoirs. The residents who live in rural district had more infection to WNV virus comparatively. About one third of participants (32.66%) stated that they keep birds at home and among them 14 (28.56%) was positive to WNV IgG antibody. The participants from Maharloo who kept birds at home comparatively had more positive cases (12.24 %) and the minimum positive cases was occur among inhabitants of Shiraz County (2.04%). However, the prevalence of positive antibodies against WNV was not significantly different among participants who kept birds (P= 0.632).The positive cases according to living in urban/rural areas have been shown in Table 5. Among all participant 42 % live in urban and 58% in rural areas whereas the positive cases were comparatively higher in the urban areas. Between all positive cases, 32.92% were urban and 21.84% in rural areas. The majority of positive cases were found in urban area of Shiraz County. The prevalence of positive cases was not statistically significantly different between rural and urban cases (P= 0.291), but it was significantly different between rural and urban cases of Shiraz County. Although the prevalence of WNV infection was relatively high, due to the similarity of symptoms to other viral diseases, health care staff had less attention to the disease.

**Table 3.** Frequency of positive West Nile virus IgG antibody according to the age groups, Fars Province, Southern Iran, 2018

**Region**	**Age (year)**	**No.**	**Positive Cases**			**Gender of positive cases**			**P-value**

			**No.**	**Percent from total (%)**	**Male**	**Percent from total (%)**	**Female**	**Percent from total (%)**	
**Shiraz**	20–30	12	0	0.00	0	0.00	0	0.00	
	31–40	23	18	12.00	10	24.39	8	19.51	
	41–50	13	9	6.00	5	12.19	4	9.76	
	51–60	8	0	0.00	0	0.00	0	0.00	
	≥60	4	0	0.00	0	0.00	0	0.00	
**Zarqan**	20–30	5	0	0.00	0	0.00	0	0.00	
	31–40	4	0	0.00	0	0.00	0	0.00	
	41–50	7	2	1.33	2	4.88	0	0.00	
	51–60	3	1	0.67	0	0.00	1	2.44	
	≥60	3	0	0.00	0	0.00	0	0.00	
**Maharloo**	20–30	5	0	0.00	0	0.00	0	0.00	
	31–40	6	2	1.33	0	0.00	2	4.87	
	41–50	3	0	0.00	0	0.00	0	0.00	
	51–60	9	2	1.33	1	2.44	1	2.44	
	≥60	1	0	0.00	0	0.00	0	0.00	
**Sepidan**	20–30	4	0	0.00	0	0.00	0	0.00	
	31–40	3	0	0.00	0	0.00	0	0.00	
	41–50	7	2	1.33	1	2.44	1	2.44	
	51–60	4	0	0.00	0	0.00	0	0.00	
	≥60	2	0	0.00	0	0.00	0	0.00	
**Marvdasht**	20–30	2	0	0.00	0	0.00	0	0.00	
	31–40	8	2	1.33	2	4.88	0	0.00	
	41–50	11	3	2.00	2	4.88	1	2.44	
	51–60	2	0	0.00	0	0.00	0	0.00	
	≥60	1	0	0.00	0	0.00	0	0.00	
**Total**	-	150	41	27.32	23	56.10	18	43.90	0.832

**Table 4.** Frequency of positive West Nile virus IgG antibody based on the history of keeping birds at home, Fars Province, Southern Iran, 2018

**Region**	**Number of samples**	**keeping birds**	**Percent from total (%)**	**Positive Cases**						**P-value**

				**Total**	**Percent from total (%)**	**Male**	**Percent from total (%)**	**Female**	**Percent from total (%)**	
**Shiraz**	60	6	4.00	1	2.04	1	7.14	0	0.00	
**Zarqan**	22	8	5.33	2	4.08	1	7.14	1	7.14	
**Maharloo**	24	17	11.33	6	12.24	2	14.29	4	28.57	
**Sepidan**	20	10	6.67	2	4.08	1	7.14	1	7.14	
**Marvdasht**	24	8	5.33	3	6.12	2	14.29	1	7.14	
**Total**	150	49	32.66	14	28.56	7	50.00	7	50.00	0.632

**Table 5.** Frequency of positive West Nile virus WNV IgG antibody according to living in urban/rural areas, Fars Province, Southern Iran, 2018

**Region**	**Number of samples**	**Urban**		**Rural**		**Positive Cases**				**Total**	**Percent from total (%)**	**P-value**

		**No.**	**Percent from total (%)**	**No.**	**Percent from total (%)**	**Urban**	**Percent from total (%)**	**Rural**	**Percent from total (%)**			
**Shiraz**	60	37	24.67	23	15.33	18	28.57	9	10.34	27	18.00	
**Zarqan**	22	8	5.33	14	9.33	1	1.59	2	2.30	3	2.00	
**Maharloo**	24	0	0.00	24	16.00	0	0.00	4	4.60	4	2.67	
**Sepidan**	20	7	4.67	13	8.67	1	1.59	1	1.15	2	1.33	
**Marvdasht**	24	11	7.33	13	8.67	2	3.17	3	3.45	5	3.33	
**Total**	150	63	42.00	87	58.00	22	34.92	19	21.84	41	27.33	0.291

## Discussion

This is the first record on WNV infection on human in Fars Province, Southern Iran. The study was conducted in five different districts and we found that the infection rate to WNV was varied based on sex and age of participants. It seems, males at middle age were more exposing to the mosquito vector bites (Table 2, 3). The statistical analysis showed that none of the demographic factors had a significant relationship with the incidence of antibodies (P> 0.05).

We found some evidence indicated that the risk of WNV infection can be age-dependent in the study areas. Similarity, a study in Ohio showed that titer of antibody against WNV in the children was 2.5 times higher than adult (33). While another study conducted on immuno-compromised and immuno-compromised patients, age was not a significant seroconversion factor. Their findings suggest that immune factors affect seroconversion (34). However, the age dependency of WNV infection is controversial.

In addition, no significant association was observed between sex and seropositivity of WNV IgG antibody among the voluntaries. Previously, equal infection of WNV was reported between both sexes in in United States (35). However, our results may indicate that both sexes had same exposure to the infected bites.

As birds are potent reservoirs of WNV, we tried to find out any correlation between keeping birds in the inhabitant houses and WNV infection rate. Noticeably, 28.56% of residents which kept birds were positive to WNV IgG antibody. Of course, these positives may be related to exposure to the virus in previous years. Generally, birds are attractive blood source for *Culex* spp (36, 37), therefore, keeping birds at home can cause distraction of mosquitoes from human to birds and thus help reduce disease transmission to human. We also found higher infection rate in urban areas comparing to rural.

Cycle of WNV in nature always occur between birds and mosquito vectors. Therefore, density of birds around inhabitants rise transmission of the disease among communities’ members in endemic area, while only 28.56 % of participants who kept birds at home were positive and we could not find any association between WNV IgG positive rate and those who kept birds at home. It likely, presence of birds around human can be good alternative blood source for the mosquito vectors. Therefore, the relationship between rate of WNV infection and keeping birds is so controversial and it is depended to situation of virus transmission in area.

West Nile Virus transmission may be varied based on rural and urban areas (38–40). It is stated that the rate of WNV infection was higher in rural areas comparing with urban areas in Bucharest and *Culex pipiens* was dominant mosquito species (41). While we also found higher positive antibody among urban residents where *Cx. pipiens* was relatively dominant (unpublished). In addition, poor drainage, catch basins, sewage, and manmade containers around houses of urban residents provide good larval development sites for *Cx. pipiens.* Moreover, poor of alternative blood source for vectors may convey the mosquito to human inhabitants in urban area.

As there is no complete cure or effective vaccine for the virus, therefore monitoring and surveillance studies regarding WNV prevalence is an important tool for health care specialists to have a future regarding this life-threatening arbovirus in order to choose the best protective plans.

As well, climate variation due to worldwide warming in recent years may increase mosquito activity period that can lead to escalating virus prevalence. Because of climate differences in Iran, the dispersal and richness of mosquito differs in different provinces of Iran. Therefore, in provinces where the weather is warmer, the life span of the mosquitoes is also increased, resulting in a higher chance of transmission. Seroepidemiological studies are necessary to define the prevalence of WNV in other parts of Iran for better decision making in the future.

## Conclusions

These results suggest circulation and exposure of the human population to WNV in south of Iran. The knowledge generated from this study will further contribute to our understanding of the ecology and epidemiology of WNV fever in Iran and will assist in the provision of public health measures to reduce the risk of exposure to the virus. It is notable that the high volume of tourist traffic in Fars Province and other economic significance of the province, awareness of the cycle of this disease is necessary and the information from this project will be used to plan the control of transmission and outbreak in Fars Province and other neighboring provinces. Previous studies have shown that horses have antibodies in the study area. The study also found that people living in the area were also in contact with the virus. Of course, the authors of this article have also researched on the probable vectors of WNV in this study area which will be published in the future. Certainly, future studies on the birds and their infection with the West Nile Virus can help to clarify the disease situation in this area.
